# How does the level of enterprise digitalization affect value creation and realization? Testing of the dual path of "based on breakthrough" and "tending to compliance"

**DOI:** 10.1371/journal.pone.0305078

**Published:** 2024-06-06

**Authors:** Jiehui Zhang, Sen Yang, Yifeng Wang

**Affiliations:** 1 Department of Financial Management, School of Economics and Management, Suqian University, Suqian City, Jiangsu Province, China; 2 Business Administration, International College, Dhurakij Pundit University, Laksi, Bangkok, Thailand; National University of Modern Languages, PAKISTAN

## Abstract

The construction of enterprise digitization serves as a "gateway" for the integration of the digital and real economies. As enterprises undergo robust digital transformations, it becomes crucial to delineate the pathway from enterprise digitization level to value creation and realization in order to maximize enterprise value. We select sample data from Chinese A-share listed companies from 2015 to 2021 as the research subject. Based on the fixed-effects model, we empirically test the impact of enterprise digitization level on both value creation and realization, as well as the mediating mechanism of entrepreneurship and internal control within it. The results indicate that the enterprise digitization level significantly enhances both value creation and realization. However, significant differences exist in the impact of the digitization level on value creation and realization among enterprises with different technological attributes and at different stages of the lifecycle. Further mechanism tests demonstrate that the "breakthrough-based" entrepreneurship and "compliance-based" internal control quality play effective mediating roles between enterprise digitization level and enterprise value. This study provides a new perspective for understanding the value creation and realization process in the digital context, and offers relevant insights for further stimulating and guiding enterprises of different types and stages to drive value enhancement with digital capabilities, thereby facilitating the deep integration of the digital with the real economy.

## Introduction

Given the continuous emergence of digital technologies such as the Internet of Things, big data, cloud computing, and artificial intelligence, the digital economy has been rapidly developing in various countries. As the leading "dual engines" in the global digital economy field, the United States and China have significant competitive advantages. According to the "Global Digital Economy White Paper (2023)," the scale of the digital economy in the United States has reached $17.2 trillion, while this scale in China is $7.5 trillion. China’s digital economy scale has grown by 10.3% year-on-year, accounting for 41.5% of GDP. Therefore, in the era of the digital economy, China’s macroeconomic development momentum has been fueled by the convergence of market traction, government impetus, technological innovations, and the driving force of the digital industry. As the primary drivers of economic development, enterprises have long deeply felt the changes brought about by this "digital force." They have actively seized strategic opportunities to embed and empower digital technologies, applying them to various aspects such as business processes, organizational conventions, resources and capabilities, and market strategies to improve enterprise performance [1]. Consequently, this effort has formed digital capabilities that drive corporate competitiveness [2], making research on enterprise digitization level the starting point for implementing and optimizing digital projects [3]. Creating value and pursuing value maximization have always been the essence of enterprise management. In the new development phase, the digital economy has given rise to a series of new business forms, technologies, and models, thereby breaking through traditional spatial and temporal limitations and promoting the cross-border allocation of factors such as capital, technology, and talent. To better achieve the maximization of shareholder value and corporate value, enterprises need to consider not only how to create value through digital opportunity perception, digital operations, and digital resource collaboration but also how to comprehensively showcase factors such as enterprise performance, culture, and strategy to the market to obtain value. Therefore, unblocking the driving transmission pathway from the enterprise digitization level to value creation and realization becomes crucial.

Enterprise digitalization is regarded as pivotal for innovation and development due to its characteristics such as availability, self-propagation, openness, and integrability [4]. However, the existence of the "information technology (IT) paradox" in the context of the widespread application of digital information technology [5–7] has led to a lack of consensus in studies on the impact of digitization on enterprises. Moreover, the literature indicates a relative scarcity of research on the endogenous factors influencing the level of enterprise digitization and its impact on value creation and realization. Therefore, there is an urgent need for theoretical research to thoroughly explore this issue from different perspectives with the aim of assisting practitioners in better understanding and bridging the gap between digitization and enterprise value. Based on the theory of value management, we investigate the impact of the enterprise digitalization level on both value creation and value realization. A heterogeneity analysis is conducted to examine the technological and lifecycle attributes of enterprises. Additionally, from the perspective of endogenous driving forces, a dual test pathway is constructed, which extends from “enterprise digitalization level—entrepreneurship—enterprise value” to “enterprise digitalization level—internal control quality—enterprise value” to analyze the mediating effects in the relationship between enterprise digitalization level and its value creation and value realization, as well as their differences. The potential marginal innovations of this paper include: (1) proposing and testing the mechanism by which the enterprise digitalization level affects value creation and realization, thereby enriching the antecedent research on enterprise value under digital contexts; (2) conducting heterogeneity research across multiple attributes to provide more targeted guidance for companies of different types and in different lifecycle stages to effectively manage their values in digital contexts; and (3) further revealing and examining the mediating effects of entrepreneurship and internal control quality with the aim of delineating the mechanism between digitalization levels and enterprise value, thus supplementing and refining relevant theoretical research on the enhancement of enterprise value in the digital economy.

## Literature review

### Research on the impact of enterprise digitalization level on enterprise value

Digitalization represents a new phase in the Information Age. In the previous literature on corporate value creation and value realization, a considerable part of the research logic was carried out in the context of information systems. Therefore, when scholars investigate the impact of corporate digitalization and its level on corporate value, they often encounter differing viewpoints similar to the "IT paradox" observed in previous perspectives. From the perspective of the resource-based theory and capability theory, some scholars point out that enterprise digitalization significantly reduces production costs and transaction costs, while also enhancing the capability for technological innovation and improvement in management models. Therefore, digitalization offers boundless potential for enhancing enterprise value [8]. Tumbas et al. (2017) [9] argued that enterprise development is significantly driven by digitalization, which promotes agility and flexibility in individuals transitioning between different work modes, thereby providing a significant boost to the enterprise value enhancement. Ren et al. (2019) [10] noted that emerging digital technologies can enhance production efficiency, increase profit income, and gain competitive advantages by influencing decision making, product design, marketing, and other aspects. From the perspective of dynamic capability theory, Tindara et al. (2022) [11] noted that digital functions such as perception, capture, integration, and interaction in internet enterprises are conducive to optimizing knowledge management methods and promoting externalized participation in open innovation, thereby ultimately enhancing organizational value cocreation. Leo (2021) [12] indicated that digitalization can help enterprises adapt to dynamic and complex internal and external environmental changes, thereby expanding the depth and breadth of enterprise value creation. However, organizational theorists argue that digital technologies have fundamentally altered the underlying logic of value creation activities within enterprises, involving systematic changes such as conceptual resetting, convention updating, process reengineering, and structural adjustments [1]. Consequently, there is a strong possibility of an insurmountable gap between the level of digitization and the existing resource capabilities of enterprises, leading to a phenomenon in which enterprise value may decline rather than increase [13, 14]. For instance, Li and Jia (2018) [15] empirically studied multiple regression methods and found that the impact of digital technology on overall corporate performance is not significant. Hajli (2015) et al. [5] used panel regression methods and discovered that the improvements in the digitalization level might only enhance the performance of some enterprises, whereas the performance of others might decline, mainly due to the high cost incurred when improving the digitalization level. AL-Adwan (2017) [16] found that the impact of the digitalization level on enterprise value may have an inverted U-shape, indicating that the value enhancement brought about by improving digitalization levels has boundaries. The review of the literature reveals that there is a relatively abundant amount of research on the relationship between digitization and firm performance, as well as firm value creation. However, a consensus has not been reached, and the literature that focuses on the impact mechanism of digitization level on enterprise value is limited. As digital technology continues to evolve and integrate further with the real economy, new characteristics of value creation and acquisition are becoming increasingly prominent [17]. Therefore, exploring how to utilize the theory of value management to link a firm’s resources and activities with value creation and realization and analyzing the relationship between digitization level and firm value, along with its transmission path, which represents an important research perspective, are crucial.

### Research on the impact of entrepreneurship on enterprise value

After years of exploration and verification, the impact of entrepreneurship on macroeconomic growth has gained relatively unanimous recognition [18]. From a microeconomic perspective, the influence of entrepreneurship on enterprises’ development and value enhancement is continually revealing new research findings. Stevenson (1985) [19] noted that entrepreneurship enables the identification of opportunities without being constrained by currently available resources, and value can be created by combining different resources to leverage and develop opportunities. Covin et al. (2019) [20] analyzed the impact of entrepreneurship on corporate value from a market perspective and found that enterprises with a higher level of entrepreneurship exhibit greater loyalty to customers, stronger abilities in exploring new markets and seizing market opportunities, and a better market reputation, which, in turn, leads to higher financial performance. Etriya et al. (2019) [21] found that entrepreneurs with more business connections, technological links, and heterogeneous networks exhibit stronger entrepreneurship, which also leads to higher financial performance for their enterprises. Niemann (2020) [22], based on survey data from 103 enterprises, discovered that entrepreneurship exerts a positive impact on environmental and corporate performance. Zhou et al. (2020) [23] used samples from non-financial sector listed companies on China’s SME board, empirically tested and found that entrepreneurship has a positive effect on corporate value. The theory of entrepreneurship posits that the entrepreneurial spirit is an essential component of enterprise capital and resources [24]. In the era of the digital economy, internet thinking is characterized by innovation, equality, and interconnection, and it thus shares fundamental similarities with entrepreneurship, and the rise of the digital economy is currently offering a new opportunity to stimulate and cultivate entrepreneurship [25]. Therefore, in the context of digitalization, exploring the role and influence of entrepreneurship in the creation of enterprise value and the realization of market value has significant implications both for both current theoretical research and economic development.

### Research on the impact of internal control quality on enterprise value

The enhancement of corporate value is closely related to the characteristics of corporate governance [26]. Internal control, as a special internal governance mechanism, provides certain supplements and refinements to the traditional corporate governance framework. Scholars have explored the relationship between internal control and enterprise value from perspectives such as internal control objectives and internal control processes. For example, Akisik et al. (2017) [27] noted that the purpose of corporate internal control is to enhance the efficiency of company operations. Therefore, a company’s internal control can effectively reflect its operational efficiency, thereby directly affecting its operational performance and ultimately affecting the company’s value. Zhou et al. (2022) [28] suggested that enterprises’ IT internal control systems based on digital platforms tend to be more intelligent and sophisticated, thus enhancing operational performance and supporting the achievement of strategic goals through control activities, information disclosure, and communication monitoring Joshi (2022) [29] analyzed a dataset of 881 global companies and found that the IT governance process capabilities (IT decision making, IT planning, IT infrastructure modernization, IT service provision, and IT monitoring) can improve IT performance, thereby enhancing business performance. However, Jarvinen et al. (2015) [30] found that if a company has significant loopholes in its internal control, its management is more motivated to engage in profit management. In contrast, for companies with high-level internal controls, management’s opportunities to engage in earnings management are generally lower than those with weak internal controls, which leads to a decline in corporate value. As digital technology becomes increasingly prevalent in enterprises, the management ideas and internal control methods endowed by digital technology are also embedded in enterprises’ daily operations, making the management processes such as finance and internal control more transparent [31]. Internal control and value management are interdependent and mutually reinforcing [32]. Based on the theory of value management, in the context of rapid development of the digital economy, what role does internal control play between the level of enterprise digitalization and enterprise value? Can it help companies optimize the allocation and utilization of information technology resources, thus assisting companies in bridging the “IT paradox” gap? This is also an important proposition addressed in this paper.

In summary, compared to the booming digital economy, relatively little research exists on the impact mechanism of corporate digitalization level on corporate value. Based on the theory of value management, we explore the impact of "breakthrough-based" entrepreneurship and "compliance-oriented" internal control governance on corporate value creation and realization under the deep integration of the digital and real economies. The objective of this paper is to supplement and refine the relevant theoretical research on enhancing corporate value through the digital economy, thereby stimulating enterprises to drive internal efficiency with digital power and achieve the goal of value management.

## Theoretical analysis and research hypotheses

### The impact of digitalization level on enterprise value

The level of digitalization can facilitate enterprises to reshape their value creation model, assisting them in capturing new profit growth points and realizing value cocreation. Based on dynamic capability theory, in the era of the digital economy, enterprises’ production and operation boundaries have been broken, the era of value creation has become outdated, and consumer-oriented, digital and nonoriented business models have gradually become a trend [33]. Digital technologies are used to provide conditions for the creation of new service-oriented business models [34]. At the same time, the cross-border platform model and online and offline interaction channels based on "integration" have also facilitated the "digital value chain" of enterprises [35]. The use of IT technology and digital tools can influence a company’s marketing management processes, helping to build closer relationships with customers and to create value for the organization [36]. A "gap" exists between enterprise value creation and enterprise value capture, however the closed loop of the business model that is rooted in the development foundation of the digital economy can assist enterprises in bridging this gap. Furthermore, through the assistance offered by the sharing and coconstruction platform constructed for the digital economy, the value creation will be enlarged, and the "life cycle" of value creation will be extended [37].

The level of digitalization can extend the processing of data from basic resources to intelligent applications and help enterprises continuously improve operational efficiency and realize value improvement. Enterprises employ data technology tools for data analysis, and dedicate high-priced labor forces to value creation [38]. This can create cost savings in the areas of searching, replication, transportation, tracking, and verification [31]. Concurrently, enterprises can further release the potential information contained in the data; transform data elements into valuable information resources; improve the level of information quality; fully mobilize knowledge and other elements; enhance the "spillover effect" of knowledge elements; realize business sharing, data sharing, and service sharing; and provide decision support across different dimensions [39]. In the process of transforming knowledge into more "intelligent" decision-making and management mechanisms, enterprises can realize the protection and cocreation of enterprise value [40]. In addition, digitalization can significantly improve the efficiency of enterprise information transmission. Through big data platforms, it can quickly present the data of various internal management and external service intelligence to stakeholders, which is conducive to the realization of enterprise value [41].

Based on the above analysis, we propose the following research hypothesis:

**H1:** *The level of enterprise digitalization has a significant positive effect on enterprise value creation and value realization*.

### The role of entrepreneurship in the relationship between the digital level and enterprise value

Entrepreneurs are the hubs that stimulate and initiate everything else [42]. They apply professional knowledge to identify important constraints, find key subjects and resources, and combine them scientifically [43]. More importantly, however, in an innovation ecological environment, their decisions are not limited to reaching solutions under given conditions to potentially overcome identified constraints [44]. In the era of the digital economy, the development of the internet has spawned a series of new business forms, new technologies and new models and has promoted the cross-border allocation of capital, technology, talent and other elements, which has provided a good opportunity for entrepreneurs to use digital technologies such as the internet, big data, cloud computing, and blockchain and has significantly promoted the "two-wheel" driving force that underlies entrepreneurship [45].

The digital economy can influence enterprise value by stimulating the entrepreneurs’ innovation passion and improving their innovation methods. As Schumpeter said, entrepreneurs have the dream and motivation to build a private kingdom. This power is extraordinary, and it is so high that entrepreneurs can quickly jump out of their own "comfort zone" to swiftly recombine resources. In the digital era, the digital innovation traits possessed by entrepreneurship have become the critical theoretical prerequisite for corporate innovation [46]. When data become a new means and key factor of production, improvements in the level of enterprise digitalization can lead entrepreneurs to accelerate the entire process of data resource transformation to data element realization and fully connect data elements to various economic activities, thus improving the efficiency of resource allocation and total factor productivity and realizing value innovation [47]. By quickly and widely tracking the technological frontier and identifying trends, such entrepreneurs can accelerate the sharing and synthesis of ideas to stimulate innovative thinking [48], optimize traditional innovation methods, improve the success rate of technological and product innovations, reshape the value creation model, and accelerate the creation and realization of enterprise value.

The level of enterprise digitalization influences enterprise value by enhancing entrepreneurial opportunities and elevating the success rate of entrepreneurship. In the early stages of entrepreneurship, the application and popularization of digital technology stimulated market vitality and consumer demand. With the improvement of the enterprise digital level, entrepreneurs can exploit the market gap, capture entrepreneurial opportunities and realize enterprise value innovation. During the entrepreneurial process, entrepreneurs can utilize the shared nature of digital economic technologies to more precisely identify potential risks, reduce internal and external information asymmetry, and utilize digital platforms to facilitate resource allocation [49] and data value, thereby enhancing the accessible interactive channels while bolstering asset flexibility [50]. In the late stage of entrepreneurship, the publicity and promotion of the digital economy platforms not only alleviate various financing constraints but also expand the effect of enterprise value realization.

In the era of the digital economy, the new business civilization system emphasizes greater openness, trust, transparency, sharing and responsibility. The entrepreneurship in this new era has gradually come to exhibit the characteristics of contracts and sharing [25]. By building a credit database, improving the new digital trust mechanism [51], and establishing a long-term and stable cooperative relationship, enterprises can accelerate the dissemination of enterprise products, improve their business image and assist in the realization of enterprise value. Subsequently, the spirit of the contract will affect internal enterprise states, guide employees to foster a sense of belonging, form a healthy and harmonious corporate culture, and improve the efficiency of value creation through improvements in the subjective initiative of the labor force.

Based on the above analysis, we propose the following research hypotheses:

**H2:** *Improving the digitalization level can stimulate entrepreneurship*, *which exerts a further significant positive effect on the creation and realization of enterprise value*.
**H2a:** *Improving the digitalization level can stimulate entrepreneurs’ innovation spirit*, *which exerts a further significant positive effect on the creation and realization of enterprise value*.
**H2b:** *Improving the digitalization level can stimulate entrepreneurs’ pioneering spirit*, *which exerts a further significant positive effect on the creation and realization of enterprise value*.
**H2c:** *Improving the digitalization level can stimulate entrepreneurs’ contract spirit*, *which exerts a further significant positive effect on the creation and realization of enterprise value*.

### The role of internal control quality in the relationship between the digital level and enterprise value

Internal control represents a set of control systems that are promoted by the enterprise management authority and engaged in by all enterprise employees. A response mechanism is thereby generated through compliance management that enhances the management and control capacity of the enterprise and thus promotes the effectiveness of its operation. The claim that solidifying the enterprise internal control system throughout the overall information system can significantly improve the quality of internal control has been widely recognized in theoretical and practical circles. With the improvement of the enterprise digitalization level, enterprise-built digital platforms, such as accounting information systems, management information systems, decision support systems and artificial intelligence systems, can automate enterprise management control, which results in decision support becoming more valuable and entrenched [28]. The development of the digital economy exerts both an agency cost effect and a debt financing effect [52]. The agency problem that currently exists between shareholders and managers can be alleviated through networked digital transmission, cloud computing, artificial intelligence and other technologies, but it can also benefit from the online transaction activities of the digital economy. Such mechanisms can provide more abundant and complete flows of information and capital for internal audits. The dual guarantee is conducive to fully leveraging the supervision function of internal audits and thereby alleviating the principal-agent problem between shareholders and managers, as well as being conducive to establishing the effective operation of the internal control system.

As a system resource and dynamic capability that forms the competitive advantage of enterprises, internal control can form sustainable competitive advantages through optimization and integration, as well as the amendment of business practices, thereby enhancing the future profitability of enterprises [53]. Internal control is embedded in the value chain of enterprises because the process of implementing control activities is, in essence, the process of value appreciation. Effective internal control also improves the transparency of accounting information disclosure [32], eases enterprise financing constraints, reduces the cost of debt financing for enterprises, boosts the formation of enterprise goodwill, and accelerates the realization of enterprise value [54].

Based on the above analysis, we propose the following research hypothesis:

**H3:** *Improving the enterprise digitalization level can further optimize the internal control quality*, *and compliance exerts a significant positive effect on the creation and realization of enterprise value*.

**Fig 1:**
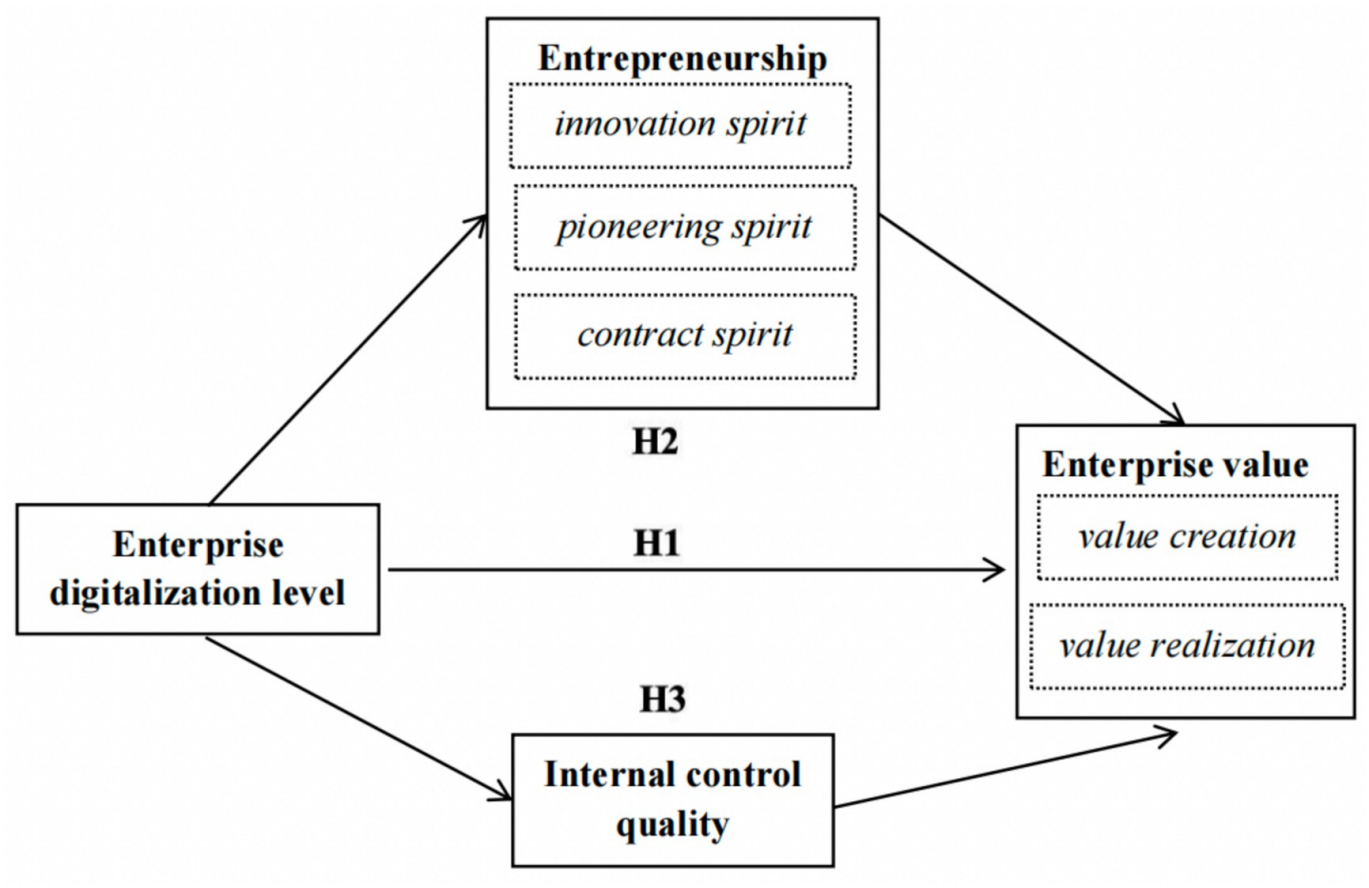
Theoretical model.

In summary, this paper constructs a theoretical model, as illustrated in Fig 1.

## Methodology

### Setting and explanation of the main variables

#### Dependent variable: Enterprise value

This paper posits that the level of digitalization in enterprises influences not only the creation of enterprise value but also its realization. Therefore, we select ROE as a measure of the level of enterprise value creation. Based on the efficient market hypothesis, a company’s market capitalization can relatively accurately reflect its true value, hence Tobin’s Q has been selected as a measure of the realization of enterprise value.

#### Independent variable: The level of enterprise digitalization

**Fig 2:**
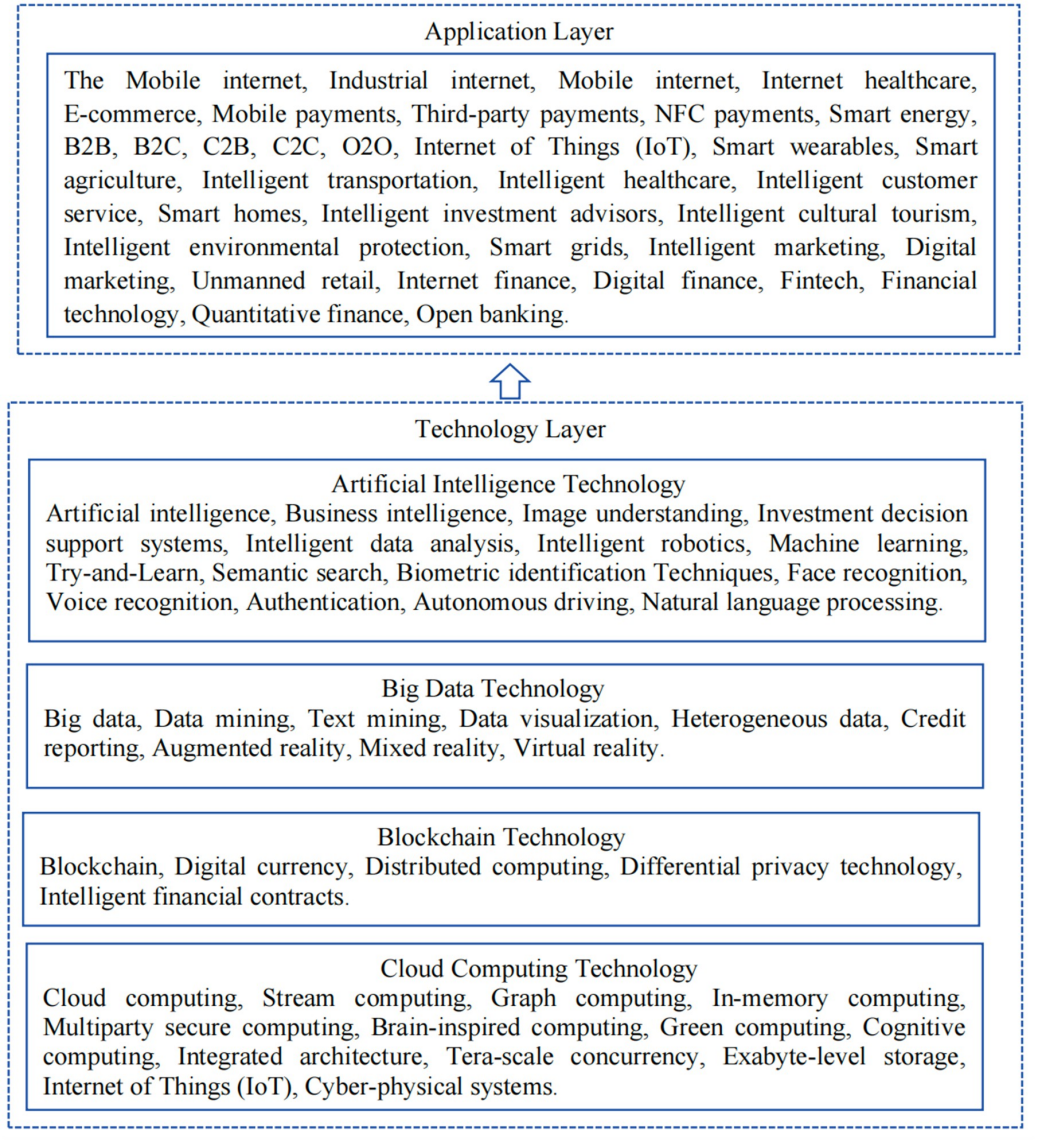
Digital keyword dictionary for enterprises.

Given that the digitalization of enterprises is a crucial strategy for their development, such characteristic information is typically reflected in companies’ annual reports, which serve as guides and summaries. Therefore, in this paper, we follow the research methods of Rippa et al. (2019) [55] and Wu et al. (2021) [56]. First, we summarize the characteristic terms of new generation information technologies, such as artificial intelligence, big data, blockchain, and cloud computing. We identify the root words of keywords from both the application and technical layers while eliminating any expressions that precede the keywords containing negative connotations. Then we use Python software to recognize and count the root words. After logarithmically processing the frequency of the word "digitalization" published in the annual reports of listed companies, we use this value as a proxy indicator of the level of digitalization in enterprises. The dictionary of digitalization keywords is shown in Fig 2.

#### Mediating variables: Entrepreneurship and internal control quality

In this paper, entrepreneurship and the internal control quality are posited to serve as important mediating variables through which the level of corporate digitalization influences value creation and realization. Drawing on the approaches employed in the literature [25, 57, 58], we focus our discussion on entrepreneurs’ innovation spirit, pioneering spirit and contract spirit. The entrepreneur’s innovation spirit can be measured by the proportion of enterprise R&D investments in the current year to the operating income, which is recorded as Esp1. The entrepreneur’s pioneering spirit can be measured by the proportion of the management’s shareholding, which is recorded as Esp2. From the perspective of employment contract spirit, the entrepreneur’s contract spirit can be measured by the wages and benefits that are paid to employees, which is recorded as Esp3. The internal control quality of enterprises is measured by the internal control index of Chinese listed enterprises issued by Dibo Enterprise Risk Management Technology Co., Ltd., and it is standardized in the regression analysis by dividing it by 100.

#### Control variables

To separate the influence of a company’s inherent financial characteristics on its value, we select factors such as firm size, growth potential, age, the asset-liability ratio, and ownership as control variables.

### Model specification

To validate the impact of a company’s digitalization level on its creation and realization of corporate value, we draw on the existing representative literature [42, 59] and establish the following benchmark regression model. In traditional linear regression models, individual differences are typically the main focus, whereas the potential impact of time and industry on the observed variables is often disregarded. This study addresses this limitation by incorporating the year as a fixed effect with the aim of mitigating the influence of time trends and seasonal variations on the research findings. This approach ensures a stronger emphasis on the long-term impact of digitalization on enterprise value without being confounded by temporal factors. Additionally, each industry possesses unique characteristics and operational dynamics, which may result in varying effects on both digitalization and enterprise value. By incorporating industry as a fixed effect, the model can estimate the relationship between digitalization levels and enterprise value more accurately, as it accounts for the heterogeneity across different industries. Therefore, to enhance the explanatory power and predictive accuracy of the model and to mitigate the potential influence of unobservable macroeconomic factors and industry-specific traits on the regression outcomes, we adopt a two-way fixed effects model incorporating both time and industry as covariates for empirical analysis.

$${\text{Value}}_{\text{i,t}}=\alpha_{0}+\alpha_{1}{\text{Szfix}}_{\text{i,t}}+\alpha_{2}{\text{Controls}}_{\text{i,t}}+\sum {\text{Year}}_{\text{t}}+\sum {\text{Ind}}_{\text{j}}+\varepsilon_{\text{i,t}}$$ (1)

In Model 1, the dependent variable is enterprise Value_i,t_, which represents the value creation or value realization of enterprise i in year t. The independent variable Szfix_i,t_ represents the digitalization level of enterprise i in year t. Year_i,t_ represents the fixed effect of the years, Ind_i,t_ signifies the fixed effect of the industry, and ε_i,t_ represents the random error.

To examine the mediating effect of entrepreneurship and internal control quality on the relationship between the level of digitalization and corporate value, we adopt the three-step mediation test method proposed by Wen et al. (2014) [60]. Building on Model 1, we further construct Models 2 and 3 as follows.


$${\text{Mediator}}_{\text{i,t}}=\beta_{0}+\beta_{1}{\text{Szfix}}_{\text{i,t}}+\beta_{2}{\text{Controls}}_{\text{i,t}}+\sum {\text{Year}}_{\text{t}}+\sum {\text{Ind}}_{\text{j}}+\varepsilon_{\text{i,t}}$$ (2)

$${\text{Value}}_{\text{i,t}}=\theta_{0}+\theta_{1}{\text{Szfix}}_{\text{i,t}}+\theta_{2}{\text{Mediator}}_{\text{i,t}}+\theta_{3}{\text{Controls}}_{\text{i,t}}+\sum {\text{Year}}_{\text{t}}+\sum {\text{Ind}}_{\text{j}}+\varepsilon_{\text{i,t}}$$ (3)

Mediator_i,t_ refers to the intermediary variable. In this paper, we note that both "breakthrough-based" entrepreneurship and "compliance-oriented" internal control quality serve as "bridges" that connect the level of digitalization to the creation and realization of corporate value. Consequently, two mediating variables, entrepreneurship Esp and internal control quality IC, are set in Models 2 and 3.

### Sample selection and data source

According to the research purpose, we select A-share listed companies in China covering the period of 2015 to 2021 as the research object and adopt the method of combining the text analysis with the financial data of the annual reports to explore the impact of the enterprise digitalization level on enterprise value creation and realization, as well as the path mechanism. The ST and *ST samples, financial industry samples, and samples exhibiting a failure to continue operations during the period or serious data loss are excluded. After the continuous variables are subjected to a bilateral 1% tail reduction, a total of 13747 groups of enterprise-year observations are finally obtained. In terms of data sources, the annual reports text information required for the digitalization level of enterprises comes from http//www.cninfo.com.cn. Other relevant financial data, such as enterprise value, mainly come from the CSMAR database. The internal control index in the third-party DIB database is used to reflect the quality of enterprise internal control. Stata 16.0 metrological analysis software is used to process and report the data.

## Empirical results and analysis

### Descriptive statistical analysis

The descriptive statistics of the research variables are presented in Table 1. The mean of ROE is 0.0947 with a standard deviation of 0.0682. The mean of Tobin’s Q is 2.6455 with a standard deviation of 1.8579, indicating a relatively significant differences in the firms’ value realization levels within the sample. The mean of the digitalization level (Szfix) is 1.6312 with a standard deviation of 1.4161. Its minimum value is 0, and the maximum value is 5.1818, suggesting a polarization in the digitalization level among the sample companies, with significant disparities. Basic statistical data on three dimensions of entrepreneurship indicate considerable variations among the sample companies, generally at a lower level. The distribution of other variables falls within a reasonable range. Furthermore, the absolute value of the kurtosis of each variable is less than 10 and the absolute value of the skewness is less than 3. Based on Kline’s (2011) [61] recommendation regarding the skewness-kurtosis rule for normal distribution, the sample data of the research variables are considered reasonably consistent with the normal distribution and are suitable for regression analysis.

**Table 1:** Descriptive statistics of the main variables.

Variable	N	Mean	Standard deviation	Min	Median	Max	Skewness	kurtosis
1.ROE	13747	0.0947	0.0682	0.0042	0.0818	0.3513	1.268	1.977
2.Tobin’s Q	13747	2.6455	1.8579	0.8354	2.0544	10.8245	2.081	5.068
3.Szfix	13747	1.6312	1.4161	0	1.3863	5.1818	0.566	-0.604
4.Esp1	13747	4.6872	4.4192	0.0300	3.71	24.7578	2.204	6.033
5.Esp2	13747	15.6523	19.9953	0	3.3422	67.9101	1.035	-0.243
6.Esp3	13747	19.5268	1.2462	17.1389	19.3802	23.3062	0.589	0.193
7.IC	13747	6.5858	0.6952	3.2428	6.6693	8.1033	-1.759	6.647
8.Size	13747	22.3412	1.2723	20.1758	22.1579	26.3596	0.799	0.543
9.Growth	13747	0.1741	0.2609	-0.1686	0.1040	1.5892	2.841	8.643
10.Age	13747	9.73	7.44	1	7	31	0.672	-0.770
11.Lev	13747	0.3969	0.1847	0.0625	0.3893	0.8259	0.211	-0.762
12.SOE	13747	0.30	0.459	0	0	1	0.867	-1.249

### Correlation analysis

The correlation test results among the variables in this study are presented in Table 2. A significant, positive correlation is observed between enterprise digitalization level and both value creation and value realization, preliminarily validating Hypothesis 1 of this study. Additionally, significant correlations are found between the enterprise digitalization level and the two mediating variables, as well as the control variables. The maximum correlation coefficient among all variables is 0.557. After excluding control variables, the maximum absolute value of the correlation coefficient is 0.342, which is less than 0.6. Referring to the criterion proposed by Zhang et al. (2016) [62], it can be preliminarily concluded that there is no multicollinearity issue in the data of this study. Furthermore, we conduct a variance inflation factor (VIF) test, obtaining VIF values for each variable ranging from 1.019 to 1.758. The VIF values of all variables are well below 10, referring to the criteria provided by Johnston (1984) [63] and Jia et al. (2018) [64], it can be concluded that there is no serious multicollinearity problem in the model.

**Table 2:** Results of correlation analysis.

	ROE	Tobin	Szfix	Esp1	Esp2	Esp3	IC	Size	Grow	Age	Lev	SOE
ROE	1											
Tobin	.280**	1										
Szf	.024**	.075**	1									
Esp1	.035**	.329**	.300**	1								
Esp2	.078**	.243**	.052**	.224**	1							
Esp3	.185**	.322**	.130**	-.145**	-.342**	1						
IC	.290**	.031**	.046**	-.050**	-0.008	.202**	1					
Size	.132**	-.417**	.027**	-.282**	-.397**	.454**	.187**	1				
Grow	.229**	.176**	.078**	.044**	.143**	-.075**	.124**	0.003	1			
Age	-.054**	-.300**	-.043**	-.245**	-.535**	.409**	-.001	.470**	-.187**	1		
Lev	-0.004	-.390**	-.021*	-.321**	-.276**	.470**	.055**	.557**	.048**	.305**	1	
SOE	-.093**	-.248**	-.095**	-.209**	-.484**	.386**	.056**	.406**	-.172**	.527**	.285**	1

Notes.

**P<0.01;

*P<0.05.

This is a Pearson two-tailed test.

### Benchmark regression results

Before conducting the benchmark regression analysis, we use the Stata command proposed by Bersvendsen and Ditzen (2021) [65] to test for potential panel slope heterogeneity issues and finding that the hypothesis of variable homogeneity holds. Furthermore, we conduct Hausman tests, and the results are presented in Table 3. In models with ROE and Tobin’s Q as dependent variables respectively, the Hausman test statistics are 117.79 and 581.52, with both p values being less than 0.01, thus rejecting the null hypothesis. This indicates a significant difference between the fixed effects model and the random effects model, thus supporting the use of the fixed effects model in this study.

**Table 3:** The benchmark regression results of the effect of digitalization level on corporate value creation and value realization.

Variable	(1)	(2)	(3)	(4)
	(ROE)	(ROE)	(Tobin’s Q)	(Tobin’s Q)
Szfix	0.0014***	0.0011***	0.0415***	0.0702***
	(2.94)	(2.77)	(3.47)	(6.58)
Size		0.0145***		-0.3470***
		(24.61)		(-25.15)
Growth		0.0571***		0.8713***
		(25.69)		(16.82)
Age		-0.0005***		-0.0188***
		(-5.52)		(-8.52)
Lev		-0.0428***		-2.2286***
		(-11.5)		(-25.66)
SOE		-0.0129***		-0.0767**
		(-8.55)		(-2.21)
Year FE	Yes	Yes	Yes	Yes
Ind FE	Yes	Yes	Yes	Yes
R^2^	0.0117	0.1078	0.1623	0.3467
N	13747	13747	13747	13747
Hausman	117.79(P<0.01)		581.52(P<0.01)	

Notes.

***p < 0.01;

**p < 0.05;

*p < 0.1.

The T-value is in parentheses.

To verify the core main effect of this paper, we apply Model (1) to test the impact of the enterprise digitalization level on enterprise value. Column (1) in Table 3 presents the regression result of the digitalization level on enterprise value creation alone, and the coefficient is significantly positive. Column (2) shows the regression result after adding the control variables, and the digitalization level coefficient remains significantly positive. Columns (3) and column (4) show the regression results of the digitalization level on the realization of enterprise value. The coefficients are both positive and both pass the significance test. The benchmark regression results show that the level of enterprise digitalization can help empower enterprises to reshape value creation and significantly improve enterprise value creation, and stakeholders have thus formed a positive expected effect, which plays a more significant positive role in terms of promoting the realization of enterprise value. The empirical results are consistent with hypothesis H1 of this paper.

### Robustness test and endogenous treatment

To improve the stability and effectiveness of the core hypothesis, a robustness test and endogenous treatment are conducted by considering the lag effect and changing the fixed effect model.

### Considering the influence of the lag effect

There is a certain time lag between the improvements of enterprises’ digitalization level and the realization of applying value creation and value capture. We lagged the enterprise value index by one period for the regression. The results are shown in Table 4. Columns (1) and (2) show the regression results for the effect of the digitalization level on the value creation and value realization of enterprises in the lagged phase. The digitalization level coefficient is significantly positive, and the adjustment R^2^ of the value realization indicator model is significantly increased, indicating that the digitalization level has a significant positive effect on the value creation and realization of enterprises in the lagged phase. The core assumption of this paper is that the H1 regression result is stable.

**Table 4:** Robustness and endogeneity test results.

Variable	Lagging by one period		Changing the fixed effect model	
	(1)	(2)	(3)	(4)
	(ROE)	(Tobin’s Q)	(ROE)	(Tobin’s Q)
L.Szfix	0.0027***	0.0785***		
	(4.56)	(6.12)		
Szfix			0.0017*	0.0342*
			(1.93)	(1.76)
Firm FE	No	No	Yes	Yes
Controls	Yes	Yes	Yes	Yes
Year FE	Yes	Yes	Yes	Yes
Ind FE	Yes	Yes	Yes	Yes
R^2^	0.0114	0.4376	0.0473	0.0230
N	8381	8381	13747	13747

Notes.

***p < 0.01;

**p < 0.05;

*p < 0.1.

The T-value is in parentheses.

#### Changing the fixed effect model

Considering that cross-sectional dependence is a critical issue and that ignoring it may lead to serious estimation bias and size distortion [66], we used the Breusch Pagan LM to test the cross-sectional correlation problem of panel data models. The statistical data of the test results rejects the null hypothesis at a 1% significance level; therefore, to account for the unobservable heterogeneity effect of individual enterprises that does not change over time, we control the individual fixed effects to reduce endogenous interference. In view of the heteroscedasticity and sequence-related problems of panel data, we conduct enterprise-level clustering for standard errors. Columns (3) and (4) in Table 4 show the regression results of the digitalization level on enterprise value creation and value realization under the stricter fixed effect model, respectively. The coefficient of the digitalization level is positive, and the significance test further supports hypothesis H1 of this paper.

### Heterogeneity test

Under the effects of the digital economy, conducting in-depth research on enterprise attributes and characteristics can improve the pertinence of management insights. This paper focuses on the technological attribute of enterprises and the attributes characteristics from the perspective of the enterprise life cycle.

#### Heterogeneity test based on the technological attributes of enterprises

With reference to the research of Ji et al. (2022) [41] and Peng et al. (2017) [67], and in accordance with the industry classification standard of the China Bureau of Statistics, the dummy variable HT is constructed to reflect the technological attributes of enterprises. High-tech enterprises are assigned a value of 1, and nonhigh-tech enterprises are assigned a value of 0. The test results from applying the two methods of group regression and interactive item processing are shown in Table 5. For value creation, columns (1) and (2) indicate that the effect of the digitalization level on non-high-tech enterprises is more prominent (β = 0.0168>β = 0.002); in addition, column (3) shows a significant negative coefficient of interaction (β = -0.0038), indicating that the technological attributes of enterprises negatively regulate the impact of the digitalization level on enterprise value creation. High-tech enterprises that are oriented toward the development and application of new technologies are facing the transformation of their fields. Under the new pattern of green and low-carbon orientation and competition, the improvement of the digitalization level exerts little effect on overcoming the bottleneck period of value creation. In contrast, nonhigh-tech enterprises, as "latecomers", accelerate the foundation of resource iteration through digital transformation, and once digital technology has been successfully implemented, this economic return value increases substantially. In terms of value realization, columns (4) and (5) show that the level of digitalization plays a more prominent role in high-tech enterprises (β = 0.1099>β = 0.0623), and the interaction coefficient of column (6) is significantly positive (β = 0.0357). The rapid innovation of high-tech enterprise products, from R&D and design to operation and sales, leverages the digital integration of value chain resources and relies on high-tech products and technologies to seize further market opportunities, thereby facilitating the shortening of the value creation to value realization cycle. Thus, the technological attributes of enterprises positively regulate the impact of the digitalization level on enterprise value realization.

**Table 5:** Heterogeneity test results based on the technological attributes.

Variable	(ROE)			(Tobin’s Q)		
	(1)	(2)	(3)	(4)	(5)	(6)
	High-tech	Non-HT	Interactive item	High-tech	Non-HT	Interactive item
Szfix	0.0020***	0.0168**	0.0018**	0.1099***	0.0623***	0.0455**
	(4.42)	(2.09)	(2.26)	(9.81)	(3.88)	(2.31)
HT			-0.0052***			0.1824***
			(2.81)			(3.99)
Szfix×HT			-0.0038***			0.0357*
			(-4.22)			(1.87)
Controls	Yes	Yes	Yes	Yes	Yes	Yes
Year FE	Yes	Yes	Yes	Yes	Yes	Yes
Ind FE	Yes	Yes	Yes	Yes	Yes	Yes
R^2^	0.1047	0.0995	0.1039	0.3348	0.3101	0.2566
N	9145	4602	13747	9145	4602	13747

Notes.

***p < 0.01;

**p < 0.05;

*p < 0.1.

The T-value is in parentheses.

#### Heterogeneity test based on the enterprise life cycle

Following the research of Li et al. (2021) [68], the enterprise life cycle is divided into the start-up period (1–6 years), growth period (7–11 years) and mature period (12 years and above) for this study, and the group regression method is used for processing. The test results are shown in Table 6. For start-ups, the level of digitalization has no significant impact on improvements in value creation, which is mainly due to the large risk coefficient of the enterprises, the obvious financing constraints, the high cost of capital in the process of improving the level of digitalization, and the small "resonance" between digitalization construction and enterprise development strategy during this period. Therefore, the enabling role of digital construction in business cannot be released in a timely manner during this period. However, during the start-up stage, enterprises can present the internal management intelligence and external service intelligence data to stakeholders, thereby leading to the phased and progressive realization of enterprise value. For growth and mature enterprises, improving the level of digitalization can have a significant positive effect on value creation and value realization. The regression coefficient shows that the level of digitalization is more significant for improving the value of growth enterprises. As the development of a growing enterprise is a dynamic and changeable process, digital resources and technical means can significantly enhance the basic operation of the enterprise, assist in the development of an intelligent operation framework to support its stronger competitiveness, facilitate easier breakthrough innovation and thus enhance the promotion of the enterprise value.

**Table 6:** Heterogeneity test results based on life cycle attributes.

Variable	(ROE)			(Tobin’s Q)		
	(1)	(2)	(3)	(4)	(5)	(6)
	start-up	growth	mature	start-up	growth	mature
Szfix	0.0001	0.0025**	0.0022**	0.0974***	0.0741***	0.0514***
	(0.2)	(2.53)	(2.41)	(5.54)	(3.74)	(3.19)
Controls	Yes	Yes	Yes	Yes	Yes	Yes
Year FE	Yes	Yes	Yes	Yes	Yes	Yes
Ind FE	Yes	Yes	Yes	Yes	Yes	Yes
R^2^	0.1487	0.1560	0.1070	0.3134	0.2885	0.2853
N	6140	2929	4678	6140	2929	4678

Notes.

***p < 0.01;

**p < 0.05;

*p < 0.1.

The T-value is in parentheses.

### Identification test of the intermediary mechanism

To clarify the path and mechanism by which the level of digitalization in enterprises affects their value, we referred to the research design of the intermediary effect test by Wen and Ye (2014) [60], which is based on the perspective of "digital level—entrepreneurship—enterprise value" and "digital level—internal control quality—enterprise value". We used Models (2) and (3) to conduct the research. Moreover, the Sobel test method was used for the robustness test.

#### The intermediary effect test based on the perspective of entrepreneurial innovation spirit

According to the empirical results displayed in Table 7, when introducing the entrepreneurial innovation spirit into the model, for value creation, the regression coefficient of the enterprise digitalization level decreases from 0.0011 to 0.0004; however, the coefficient of the entrepreneurial innovation spirit on value creation is not significant. For value realization, the regression coefficient of enterprise digitalization level decreases from 0.0702 to 0.0296, and the regression coefficient of entrepreneurial innovation spirit on value realization is significant (β = 0.0784). Therefore, improving the level of enterprise digitalization can significantly stimulate entrepreneurial innovation spirit, thereby driving the realization of enterprise value. In the context of entrepreneurial sharing in the digital economy, the knowledge spillover effects stimulate innovative thinking; but there may be certain switching costs when tracking technological frontiers and optimizing traditional innovation methods. So that the mediating effect of entrepreneurial innovation spirit on the relationship between the digitalization level and value creation is not obvious. However, driven by the spirit of entrepreneurial innovation, enterprises in an open innovation environment have delivered a continuously updated and iterative innovation model to stakeholders. The cross-industry and cross-regional "breakthrough" innovation cooperation further releases positive signals to the capital market and promotes the realization of enterprise value. The empirical data show that entrepreneurial innovation spirit plays a mediating role between the realization of enterprise value and the digitalization level, which provides some empirical evidence supporting hypothesis H2a.

**Table 7:** Test results of the intermediary effect based on entrepreneurs’ innovation spirit.

Variable	(ROE)			(Tobin’s Q)		
	(1)	(2)	(3)	(4)	(5)	(6)
	ROE	Esp1	ROE	Tobin’s Q	Esp1	Tobin’s Q
Szfix	0.0011***	0.4805***	0.0004***	0.0702***	0.4805***	0.0296***
	(2.83)	(17.96)	(6.14)	(6.58)	(17.96)	(2.79)
Esp1			0.0009			0.0784***
			(0.76)			(22.63)
Controls	Yes	Yes	Yes	Yes	Yes	Yes
Year FE	Yes	Yes	Yes	Yes	Yes	Yes
Ind FE	Yes	Yes	Yes	Yes	Yes	Yes
R^2^	0.1078	0.3227	0.1101	0.3467	0.3227	0.3691
N	13747	13747	13747	13747	13747	13747
Sobel test	4.3656			15.138***		
	Mechanism interrupted			Effective mechanism—forward		
				conduction mechanism		

Notes.

***p < 0.01;

**p < 0.05;

*p < 0.1.

The T-value is in parentheses.

#### The intermediary effect test based on the perspective of entrepreneurial pioneering spirit

As shown in Table 8, when introducing the entrepreneurial pioneering spirit into the model, the regression coefficient of the digitalization level on entrepreneurial pioneering spirit is significantly positive (β = 0.7320). Furthermore, the regression coefficient of the level of digitalization in enterprises on value creation decreases from 0.0011 to 0.0009, and that on value realization decreases from 0.0702 to 0.0700, both of which are significant. Therefore, we can infer that the level of enterprise digitalization can effectively improve the entrepreneurial pioneering spirit of entrepreneurs, and this positive driving effect is further transmitted to enterprise value creation and value realization. Entrepreneurs use digital platforms to find market gaps, capture potential entrepreneurial opportunities, reduce entrepreneurial risks, achieve breakthroughs and expand their social networks of entrepreneurs. The intermediary role of pioneering spirit provides further evidence to support hypothesis H2b in this paper.

**Table 8:** Test results of the intermediary effect based on pioneering spirit.

Variable	(ROE)			(Tobin’s Q)		
	(1)	(2)	(3)	(4)	(5)	(6)
	ROE	Esp2	ROE	Tobin’s Q	Esp2	Tobin’s Q
Szfix	0.0011***	0.7320***	0.0009**	0.0702***	0.7320***	0.0700***
	(2.83)	(6.09)	(2.30)	(6.58)	(6.09)	(6.58)
Esp2			0.0003***			0.0030***
			(9.00)			(3.67)
Controls	Yes	Yes	Yes	Yes	Yes	Yes
Year FE	Yes	Yes	Yes	Yes	Yes	Yes
Ind FE	Yes	Yes	Yes	Yes	Yes	Yes
R^2^	0.1078	0.2870	0.1102	0.3467	0.2870	0.3462
N	13747	13747	13747	13747	13747	13747
Sobel test	5.1981***			3.1924***		
	Effective mechanism—forward			Effective mechanism—forward		
	conduction mechanism			conduction mechanism		

Notes.

***p < 0.01;

**p < 0.05;

*p < 0.1.

The T-value is in parentheses.

#### The intermediary effect test based on the perspective of entrepreneurial contract spirit

According to the empirical results shown in Table 9, when introducing the entrepreneurial contract spirit into the model, the regression coefficient of digitalization level on entrepreneurial contract spirit is significantly positive (β = 0.0947). Furthermore, the regression coefficient of the enterprise digitalization level on value creation decreases from 0.0011 to 0.0002, and that on value realization decreases from 0.0702 to 0.0420, both of which are significant. Therefore, we can infer that the level of enterprise digitalization contributes to the establishment of an entrepreneurial contract spirit and thus has a positive driving effect on enterprise value creation and value realization. Under the new digital trust mechanism, the entrepreneurs’ contract spirit can be widely recognized by society for creating a healthy and harmonious organizational system, providing a fair and honest corporate culture, and improving the efficiency of organizational operations. The above empirical results offer support for H2c.

**Table 9:** Test results of the intermediary effect based on entrepreneurial contract spirit.

Variable	(ROE)			(Tobin’s Q)		
	(1)	(2)	(3)	(4)	(5)	(6)
	ROE	Esp3	ROE	Tobin’s Q	Esp3	Tobin’s Q
Szfix	0.0011***	0.0947***	0.0002***	0.0702***	0.0947***	0.0420***
	(2.83)	(22.33)	(6.13)	(6.58)	(22.33)	(3.89)
Esp3			0.0208***			0.2977***
			(22.99)			(13.95)
Controls	Yes	Yes	Yes	Yes	Yes	Yes
Year FE	Yes	Yes	Yes	Yes	Yes	Yes
Ind FE	Yes	Yes	Yes	Yes	Yes	Yes
R^2^	0.1078	0.7705	0.1408	0.3467	0.7705	0.3547
N	13747	13747	13747	13747	13747	13747
Sobel test	16.1391***			11.8794***		
	Effective mechanism—forward			Effective mechanism—forward		
	conduction mechanism			conduction mechanism		

Notes.

***p < 0.01;

**p < 0.05;

*p < 0.1.

The T-value is in parentheses.

#### The intermediary effect test based on the perspective of internal control quality

We extend the analysis from the perspective of "entrepreneurship" to the perspective of "internal control". Table 10 shows the results of incorporating the internal control quality into the model. The regression coefficient of digitalization level on internal control quality is significantly positive (β = 0.0202). Furthermore, the regression coefficient of a company’s digitalization level on value creation decreases from 0.0011 to 0.0004, and the regression coefficient on value realization decreases from 0.0702 to 0.0663. All of these coefficients pass the significance tests, indicating that the enterprise digitalization level can significantly improve enterprise internal control quality. Enterprises will generate response mechanisms based on compliance management concepts, thereby forming institutional resources and dynamic capabilities for competitive advantages. This will also contribute to the formation of corporate goodwill, thus promoting value creation and realization. The above empirical results offer support for H3 in this paper.

**Table 10:** Test results of the intermediary effect based on the perspective of internal control quality.

Variable	(ROE)			(Tobin’s Q)		
	(1)	(2)	(3)	(4)	(5)	(6)
	ROE	IC	ROE	Tobin’s Q	IC	Tobin’s Q
Szfix	0.0011***	0.0202***	0.0004**	0.0702***	0.0202***	0.0663***
	(2.83)	(4.23)	(2.28)	(6.58)	(4.23)	(6.22)
IC			0.0288***			0.1976***
			(36.01)			(10.39)
Controls	Yes	Yes	Yes	Yes	Yes	Yes
Year FE	Yes	Yes	Yes	Yes	Yes	Yes
Ind FE	Yes	Yes	Yes	Yes	Yes	Yes
R^2^	0.1078	0.2662	0.1101	0.3467	0.2662	0.3505
N	13747	13747	13747	13747	13747	13747
Sobel test	4.1800***			3.9011***		
	Effective mechanism—forward			Effective mechanism—forward		
	conduction mechanism			conduction mechanism		

Notes.

***p < 0.01;

**p < 0.05;

*p < 0.1.

The T-value is in parentheses.

## Conclusion and implications

### Discussion of findings

Value creation and realization have always been important goals for microenterprises. In the context of the digital economy, significant changes have occurred in both market and consumer behavior. Digital transformation has become an inevitable trend; however, many enterprises still face confusion during the transformation process. Does continuously improving the level of enterprise digitalization contribute to value creation and realization? What is the driving transmission path extending from the digital level to value creation and value realization? In this paper, we use China’s A-share listed companies as the research object, and the method of combining the text analysis of the annual reports with their financial data is used to explore the impact of the enterprise digitalization level on enterprise value creation and value realization, as well as the path mechanism underlying it. We obtain several interesting results.

First, a benchmark regression on the impact of the digitalization level on enterprise value creation and value realization is performed. The empirical results show that the enterprise digitalization level can significantly improve the level of enterprise value creation and exert a more significant positive effect on enterprise value realization. At the same time, the lag effect is considered in this paper and the fixed effect model is changed as a robustness test and endogenous treatment. At last, the empirical results show that the main effect research conclusions are more robust and reliable.

Second, based on the consideration of asymmetric effects, we conducted heterogeneity analysis and tests on the samples. Research has shown that the level of digitalization plays a more prominent role in the value creation of nonhigh-tech enterprises than in that of high-tech enterprises; moreover, regarding value realization, the digital level plays a more prominent role in high-tech enterprises than in nonhigh-tech enterprises. From the perspective of the enterprise life cycle, the level of digitalization had no significant impact on the improvements in value creation for start-ups but can significantly promote the realization of enterprise value. For growing and mature enterprises, improving the level of digitalization has a significant positive effect on value creation and value realization, and the level of digitalization is more significant for improving the value of growing enterprises.

Finally, based on the channel mechanism test, we find that affected by conversion costs and other factors, except for the insignificant mediating effect of corporate innovation spirit in digitalization level and value creation, the empirical results show that the improvement of corporate digitalization level can effectively stimulate various dimensions of entrepreneurship; At the same time, such improvement can also promote the internal control quality in enterprises and generate a response management mechanism based on compliance. The improvement of these factors is conducive to the creation and realization of enterprise value.

### Implications and suggestions

Based on the above findings, from the perspective of practice, this study serves to provide beneficial suggestions for managers of the firms as well as policymakers of the governments with the aim of stimulating the internal governance efficiency of enterprises through digital power and then promoting the deep integration of the digital economy and the real economies.

First, the government should provide more effective support and guidance for the improvement of enterprise digitalization. On the one hand, the government can increase investments in digital infrastructure construction and security, including network infrastructure, data centers, and cloud computing platforms, to provide a favorable digital environment and conditions for enterprises. Additionally, the government can formulate personalized support policies and measures based on the digitalization needs and characteristics of different types of enterprises. For non-high-tech enterprises, more digital training and technical support can be provided to help them enhance their digital capabilities and promote value creation. For high-tech enterprises, increased investment and policy support can encourage them to accelerate digital transformation and enhance value realization capabilities. Start-up enterprises, especially, require the government to provide an inclusive, agile, and low-cost "ticket" for digital transformation. On the other hand, the government should also strengthen its guidance on entrepreneurship and the regulation of internal control quality within enterprises. By providing entrepreneurial policy support, entrepreneurship education and training, and mentorship, the government can actively create an environment and atmosphere conducive to innovation, stimulating entrepreneurship. Furthermore, the government should strengthen the supervision of internal control quality within enterprises, establish sound internal control systems and audit supervision mechanisms, standardize internal operations and management behavior, enhance operational transparency and compliance, and ultimately promote sustained growth of enterprise value.

Second, enterprise managers should formulate a digital strategy that aligns with the actual situation of the enterprise and accounts for the industry and lifecycle status of the business. The findings of this research show that, in terms of value creation, the level of digitalization plays a more prominent role for non-high-tech enterprises than for high-tech enterprises. Conversely, regarding value realization, the impact is more pronounced for high-tech companies. Therefore, managers need to invest in digital resources in a targeted manner, considering the actual situation of the enterprise, the characteristics of the industry, and the market development trends. For start-ups, effective planning of fixed assets investments and strengthening the organic and flexible integration of existing digital technologies are advisable in business operations such as research and development, production, and marketing. For enterprises in the growth and mature stages, from the perspective of enhancing corporate value objectives, the investment and application of digital technologies such as 5G, blockchain, big data, artificial intelligence, and cloud computing should potentially increase. Furthermore, by integrating platforms such as digital ecosystems, enterprises can achieve complementary advantages and resource sharing, thereby enhancing their corporate value.

Third, enterprises should pay attention to the new developments of entrepreneurship in the digital context and cultivate and transmit entrepreneurship from the perspective of internal and external motivations. In the era of the digital economy, information is spreading rapidly. In this new business civilization system, a new digital trust mechanism has been formed between enterprises and society. Therefore, enterprises should not only cultivate innovation and entrepreneurship on the foundation of innovation, equality and interconnection but also strive to fulfill their commitments to stakeholders based on the perspective of internal and external sustainable development motivations, for example, voluntarily expending extra time and effort to ensure the performance of the transaction contract. They should operate with integrity and adherence to digital governance norms, embedding entrepreneurship into corporate culture and disseminating it to the market. And in turn, the market’s ability to screen enterprises can be enhanced to allow improvements in the digital level to continue to effectively drive enterprise value creation and realization.

Finally, enterprises should build an internal control implementation system that matches their digital strategy and elucidate the mechanism transmission path driven by internal governance effectiveness. In the digital construction process, enterprises are easily affected by path dependence, and a gap exists between existing resource conditions and the management capacity base. Therefore, enterprises should adjust and build an internal control implementation system that matches the digital strategy in a timely manner and improve the internal governance structure and dynamic organizational structure. Then, enterprises can implement internal control by applying engineering thinking, select internal control evaluation indicators that are adapted to the digital situation, and build a compliant digital management response mechanism to help transcend the "digital paradox" and realize the improvement of internal governance efficiency that drives enterprise value.

### Limitations and directions of future research

In the previous literature, the relationships among enterprise digitalization level, entrepreneurship, and internal control quality have rarely been studied. We have explored the role and mechanism of these two internal characteristic factors in the process through which the enterprise digitalization level enhances value. However, we have not analyzed the noneconomic effects of these two factors on enterprises in the context of digitalization, nor have we considered whether the impact of digitalization on enterprise value may vary under the dynamic external environmental conditions. These limitations offer a promising direction for further investigation.

## Supporting information

S1 Data (XLSX)
